# Feedback in tropical forests of the Anthropocene

**DOI:** 10.1111/gcb.16293

**Published:** 2022-06-30

**Authors:** Bernardo M. Flores, Arie Staal

**Affiliations:** ^1^ Graduate Program in Ecology Federal University of Santa Catarina Florianopolis Brazil; ^2^ Copernicus Institute of Sustainable Development Utrecht University Utrecht The Netherlands

**Keywords:** Amazon forest, Anthropocene, climate change, Congo forest, resilience, Social–ecological systems, Southeast Asian forests

## Abstract

Tropical forests are complex systems containing myriad interactions and feedbacks with their biotic and abiotic environments, but as the world changes fast, the future of these ecosystems becomes increasingly uncertain. In particular, global stressors may unbalance the feedbacks that stabilize tropical forests, allowing other feedbacks to propel undesired changes in the whole ecosystem. Here, we review the scientific literature across various fields, compiling known interactions of tropical forests with their environment, including the global climate, rainfall, aerosols, fire, soils, fauna, and human activities. We identify 170 individual interactions among 32 elements that we present as a global tropical forest network, including countless feedback loops that may emerge from different combinations of interactions. We illustrate our findings with three cases involving urgent sustainability issues: (1) wildfires in wetlands of South America; (2) forest encroachment in African savanna landscapes; and (3) synergistic threats to the peatland forests of Borneo. Our findings reveal an unexplored world of feedbacks that shape the dynamics of tropical forests. The interactions and feedbacks identified here can guide future qualitative and quantitative research on the complexities of tropical forests, allowing societies to manage the nonlinear responses of these ecosystems in the Anthropocene.

## Graphical abstract

Tropical forests are complex systems containing myriad interactions and feedbacks with their biotic and abiotic environments, but as the world changes fast, the future of these ecosystems becomes increasingly uncertain. Our findings reveal an unexplored world of feedbacks that shape the dynamics of tropical forests. The interactions and feedbacks identified can guide future qualitative and quantitative research on the complexities of tropical forests, allowing societies to manage the nonlinear responses of these ecosystems in the Anthropocene.

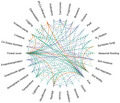

## 1 INTRODUCTION

Forests across the tropics (30°N−30°S) are crucial for the functioning of the Earth system by storing vast amounts of carbon (Bonan, 2008; Mitchard, 2018), for global ecology by harboring much of the planet's terrestrial biodiversity (Barlow et al., 2018), and for humans' livelihoods by providing a range of products and many other ecosystem services (IPBES, 2019; Levis, Flores, et al., 2020). However, tropical forests are under increasing pressure from deforestation, fires, and other global‐climate‐change‐induced disturbances (Edwards et al., 2019; Malhi et al., 2008). Feedback mediates the effects of stochastic perturbations and stressing conditions on the functioning and distributions of tropical forests. Positive feedback amplifies change, whereas negative feedback dampens it (DeAngelis et al., 1986). By amplifying change, positive feedback in particular may propel ecosystems into entirely contrasting regimes (Scheffer, 2009). Visualizing feedback in tropical forests is therefore a key step toward understanding how these ecosystems might respond to global changes. For example, tropical forests have been acting as a net carbon sink, absorbing anthropogenic CO_2_ emissions, but they can do that only up until a certain level of CO_2_‐induced global climate change, after which they may become a net source, owing to combinations of increased mortality, respiration and fires, and reduced growth (Clark, 2004; Covey et al., 2021; Cuni‐Sanchez et al., 2021; Gatti et al., 2021; Hubau et al., 2020; Mitchard, 2018). The combination of interacting processes like these makes tropical forests very complex systems; understanding their feedbacks is therefore paramount for both the global and local societies if they wish to preserve these ecosystems and manage them sustainably (IPCC, 2021; Scheffer et al., 2015; Steffen et al., 2018).

**FIGURE 1:**
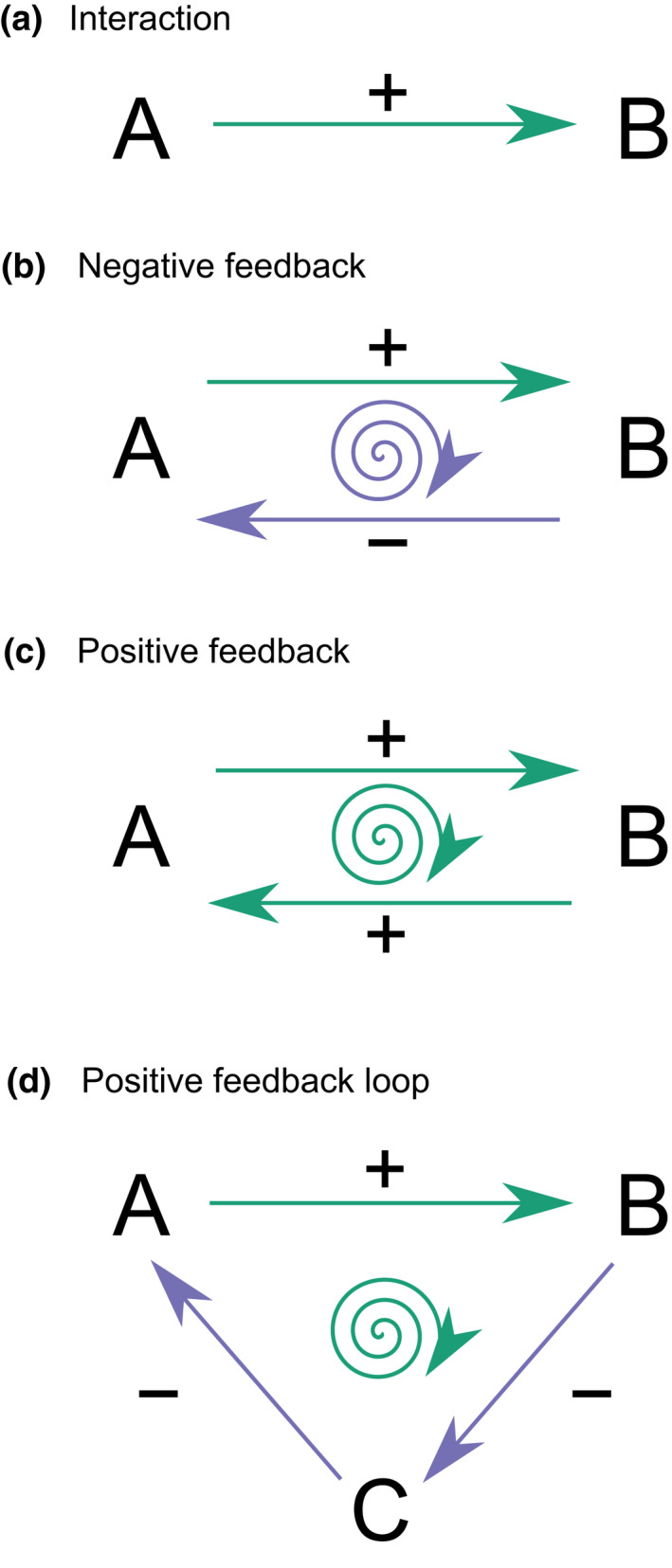
The concepts of *interaction*, *feedback*, and *feedback loop*. (a) *Interaction* is a one‐way cause‐and‐effect relation between two components, here shown as A and B. (b) *Negative feedback* occurs when two reciprocal cause and effect interactions, a positive and a negative, result in a negative net effect on both components that dampen change in the system. (b) *Positive feedback* occurs when two reciprocal positive interactions result in a positive net effect on both components that amplifies change in the system. (d) *Feedback loop* is a feedback involving three or more interacting components, here illustrated by an example resulting in a positive net effect. A *feedback loop* will be positive or negative depending on the combination of signs of the interactions.

We will refer to *interactions* as one‐way cause‐and‐effect relations between two components in a system, to *feedback* as the phenomenon of reciprocal cause and effect, and to *feedbacks* as particular cause‐and‐effect relations. *Feedback* involves two interacting components, whereas a *feedback loop* involves three or more interacting components in which a cause feeds back to itself. A feedback will be positive or negative depending on the sign of the combined interactions. The concepts of positive and negative feedback are illustrated in Figure 1. In complex natural and social systems, the combined effect of various feedbacks determines to which extent shocks and pressures are being absorbed by the ecosystem (Scheffer, 2009). In this review, we search across the scientific literature for evidence of interactions in tropical forests that may form feedbacks and feedback loops. The main focus will be at processes involving the main structure of the ecosystem: forest cover. Although non‐exhaustive, our review covers different research fields, and a large range of spatial scales from global to local. By qualitatively connecting separate cause–effect relations published in the peer‐reviewed literature from different continents into a single network, we generate new hypotheses on the existence of previously unidentified feedback loops in tropical forests of the Anthropocene.

## 2 TROPICAL FOREST INTERACTIONS

### 2.1 Interactions with the global climate

On the scale of the Earth system, tropical forests interact with the global climate. Forests across the tropics store between 200 and 300 Gt C (Mitchard, 2018). If this carbon would enter the atmosphere, CO_2_ concentrations would rise by more than 100 ppm (Friedlingstein et al., 2019), exacerbating global warming (IPCC, 2021), which reduces the tropical carbon sink (Sullivan et al., 2020); but not only temperature, also rainfall patterns would be affected by global climate change. Drought can cause widespread tree mortality (McDowell et al., 2018), productivity loss (Xu et al., 2019), and species turnover (Esquivel‐Muelbert et al., 2019), and eventually turn tropical forests from net carbon sinks into carbon sources (Hubau et al., 2020). Global climate models indicate that severe global warming would result in a contraction of suitable area for tropical forests in the Amazon, although it could at least be partly compensated by an expansion in Africa (Staal, Fetzer, et al., 2020; Zelazowski et al., 2011). Also, such increases in atmospheric CO_2_ concentrations might benefit remaining forests through “CO_2_ fertilization” (Norby et al., 2005), which would enhance trees' water‐use efficiency (Cernusak et al., 2019; but see Bauters et al., 2020), although it may not result in a net increase in tree growth (Peñuelas et al., 2011; Van der Sleen et al., 2015). Thus, the net global cooling effect of tropical forests through carbon storage feeds back in contrasting ways and differs among continents. Although different climate models produce different results, the global‐scale forest‐carbon feedback, considered in isolation, may be close to neutral (Huntingford et al., 2013). Tropical forests warm the atmosphere by reducing the albedo (reflectivity) of the land surface, but this is more than compensated by their cooling effect through latent heat release related to evapotranspiration (Bonan, 2008; Longo et al., 2020). In addition, the “vapor buoyancy feedback” is a recently proposed negative climate feedback that may play a role in temperature regulation by forest cover. The rise of light, moist air would be compensated by warming of dry air, which increases outgoing longwave radiation and thus leads to atmospheric cooling. This cooling becomes stronger with climate warming, because warmer atmospheres contain more moisture. This negative feedback would partially compensate the atmospheric moisture–temperature positive feedback (Seidel & Yang, 2020).

Evidence from the Amazon points at other feedbacks between the forest and global climate. Not only droughts, but also storms can cause significant tree mortality (Aleixo et al., 2019). However, there are two important differences between both disturbances: first, the spatial scale of the so‐called “windthrows” tends to be smaller than that of droughts, but locally more destructive (Negrón‐Juárez et al., 2010); and second, they are associated with severely wet rather than dry periods (Aleixo et al., 2019). This may be one of the several mechanisms by which increasing interannual rainfall variability negatively affects forest cover in the wet tropics (Holmgren et al., 2013). Extremely wet years including windthrows depend on sea surface temperatures and are, consistent with global climate change, on the rise (Haylock et al., 2006). Further adding to the complexity involving tropical windthrows is that they enhance soil organic carbon content (Dos Santos et al., 2016).

One type of tropical forest to highlight in relation to the global climate is peat forest. Up to recently, it was believed that these mainly occurred in Indonesia (Page et al., 2011), but it is now recognized that they exist in the Amazon and Congo forests as well (Dargie et al., 2017; Draper et al., 2014). Although they span only 5% of tropical forests, the peat underneath them contains around 70−130 Gt C (Mitchard, 2018). Peat formation accumulates faster at high temperature (Fischer et al., 2018).

Although interactions with the global climate apply to the global scale by definition, some important processes occur at local to regional scales. This includes the change in albedo by forests, but also global climate change feeds back to affect local‐scale processes in tropical forests such as deforestation, fires and windthrows. This highlights the complex interplay between tropical forests and the Earth System.

### 2.2 Interactions with rainfall

It was recognized already by Von Humboldt and Bonpland (1807) that tropical forests depend on high rainfall levels, and also the idea that rainfall levels depend on forests can be traced back to at least the 19th century (Bennett & Barton, 2018). More recently, we have gained detailed insight in how tropical forests enhance atmospheric moisture content and subsequently enhance regional rainfall levels (Spracklen et al., 2018). This occurs both through transpiration, the process by which trees extract moisture from the soil and release that to the atmosphere through their leaves (Aragão, 2012), and by interception evaporation, which is the evaporation from wet leaf surfaces that directly results from rainfall (Miralles et al., 2010). Because transpiration involves moisture stored belowground, it can elevate rainfall levels on seasonal time scales (Staal, Tuinenburg, et al., 2018), whereas interception evaporation returns moisture to the atmosphere within hours of a rainfall event (Wang‐Erlandsson et al., 2014). The atmospheric residence time of this moisture is around 10 days (Spracklen et al., 2012; Van der Ent & Tuinenburg, 2017) and the median distance of transport before the next rain event was simulated to be 600 km in the Amazon (Staal, Tuinenburg, et al., 2018). Because this distance is exceeded by the size of the forest, a single water molecule can rain out several times before leaving the system (Salati et al., 1979; Staal, Tuinenburg, et al., 2018; Zemp et al., 2014). Partly because of this cascading moisture recycling, forests alleviate droughts (O'Connor et al., 2021; Staal, Tuinenburg, et al., 2018). Not just warmer, but especially drier atmospheres enhance evapotranspiration (Smith et al., 2020), and droughts increase tree mortality (Allen et al., 2010; Bonal et al., 2016), impede growth (Yuan et al., 2019), and enhance fires (Aragão et al., 2018). Furthermore, atmospheric moisture increases forest cover stability in cloud forests during dry periods (Urrego et al., 2010). The forest‐rainfall feedback is of large significance for the stability of especially the Amazon forest system (Zemp et al., 2017) and may even be underestimated due to the generally overlooked nonlinear effect of atmospheric moisture on rainfall events (Baudena et al., 2021). Also contributing to forest‐rainfall feedback is the positive effect of evapotranspiration by forests on monsoons, thereby increasing moisture inflow from the oceans. The increased evapotranspiration implies enhanced latent heat release at the surface, which is released in the lower atmosphere as the moisture condensates. This increases the heating gradient between the land and the atmosphere, leading to a larger moisture influx during monsoon seasons (Boers et al., 2017; Levermann et al., 2009). Another proposed mechanism by which forest‐induced evapotranspiration increases incoming moisture fluxes is not through the enhancement of temperature gradients but through a drop in pressure as moist air rises and condensates. This mechanism would pull in more moist air from lower altitudes, thus creating a “biotic pump,” a positive forest‐rainfall feedback that is sustained as long as sufficient moisture is supplied by the forest (Makarieva & Gorshkov, 2007; Sheil, 2018).

The story of forest‐rainfall feedback further complicates at smaller spatial scales. First, forest loss may locally (~10 km) increase rainfall: heterogeneous forest cover increases surface roughness, which increases local atmospheric turbulence and rainfall (Lawrence & Vandecar, 2015). Thus, although forests have a net positive effect on rainfall at regional scales, generating a positive feedback, the opposite appears to be true at local scales. Second, clouds generated by atmospheric moisture enhancement reduce solar irradiation, possibly limiting photosynthesis (Nemani et al., 2003). Indeed, a greening of the Amazon during drought has been reported and attributed to this mechanism (Huete et al., 2006; Saleska et al., 2007), but also heavily disputed (Morton et al., 2014; Samanta et al., 2010). Tropical forest tree communities are likely adapted to irradiation seasonality, producing new green leaves in the dry season, increasing their photosynthetic efficiency, and producing a greening pattern visible from space (Wu et al., 2016).

### 2.3 Interactions with aerosols

Trees produce volatile organic compounds (VOCs) which are converted in the atmosphere into secondary aerosols. There, they act as cloud condensation nuclei and promote the occurrence of rainfall (Pöschl et al., 2010). Furthermore, these aerosols diffuse solar radiation, allowing it to reach deeper canopy layers than would have otherwise been the case. The resulting increase in photosynthesis has been calculated for tropical forests to be considerably larger than the photosynthetic cost to the trees (Rap et al., 2018), suggesting that enhanced photosynthetic capacity leading to the production of VOCs positively feeds back to photosynthetic capacity. VOC emissions are measured to be higher in dry seasons in the Amazon than in the wet seasons (Nölscher et al., 2016) and aerosols are removed from the atmosphere by rainfall (Lohmann & Feichter, 2005).

Irradiation warms the Earth's surface and thereby its atmosphere (IPCC, 2021; Rasool & Schneider, 1971), and aerosols are long known to have a surface cooling effect via a decrease in incoming radiation (Rasool & Schneider, 1971). However, they affect monsoon dynamics as well, as is illustrated by the inferred relation between the South Asian monsoon and aerosol loading (Li et al., 2016). Through their cooling effect, aerosols reduce the heating gradient between the land and atmosphere (Ramanathan et al., 2001). Indeed, 20th century weakening of the South Asian monsoon can be explained by increased Anthropogenic aerosol loading (Bollasina et al., 2011), highlighting the potentially large effects of aerosols on regional climates.

In addition to biogenic aerosols, also pyrogenic aerosols are found in the atmosphere above tropical forests (Echalar et al., 1995). The effects of these aerosols on atmospheric dynamics are complex (Liu et al., 2020). For example, they can act as cloud condensation nuclei, thus reducing atmospheric vapor content (Crutzen & Andreae, 1990). In particular, smoke particles may decrease droplet size, causing clouds to form at higher altitudes, which releases latent heat there (Andreae et al., 2004). Smoke also absorbs sunlight, reducing irradiation at the land surface, negatively affecting evapotranspiration and consequently atmospheric moisture content (Koren et al., 2004). However, black carbon, an aerosol uniquely resulting from biomass burning, absorbs solar radiation and may inhibit cloud formation (Hodnebrog et al., 2014). Due to such complexities involving cloud formation and radiation effects, the effects of fire on rainfall have been reported to have opposing sign depending on time of day (Liu et al., 2020). Furthermore, fires indirectly generate ozone, which is estimated to have a larger inhibiting effect on primary productivity than the photosynthesis‐enhancing effect of the aerosols through the diffusion of light (Yue & Unger, 2018).

In short, there is observational evidence of multiple positive and negative feedbacks in tropical forests that involve aerosols. These feedbacks operate at landscape to regional scales. Although empirical evidence of (at least) individual interactions is accumulating, many uncertainties still exist.

### 2.4 Interactions with fire

A reciprocal negative effect between forest cover and fire occurrence creates a positive feedback that is believed to cause alternative stable states in tropical ecosystems (de Dantas et al., 2016; Hirota et al., 2011; Pausas & Bond, 2020; Pausas & Dantas, 2017; Staal, van Nes, et al., 2018; Staver et al., 2011; Van Nes et al., 2018). Open landscapes such as savannas and grasslands contain much C4 grass biomass, which becomes flammable when it dries out (Kelley et al., 2019). The resulting wildfires contribute to maintain landscapes with low tree cover (Staal, van Nes, et al., 2018) and patchy C4 grass cover (Bond & Keeley, 2005). Furthermore, forest cover creates shady, humid and cooler microclimatic conditions beneath the canopy, inhibiting grasses and fires simultaneously (De Frenne et al., 2021; Longo et al., 2020; Murphy & Bowman, 2012; Uhl & Kauffman, 1990). Thus, both low‐ and high‐forest cover ecosystems across the tropics are self‐stabilizing, except under very dry or very wet conditions, where only one ecosystem state is stable: low forest cover in the dry regions and high forest cover in the wet regions (Hirota et al., 2011; Kelley et al., 2019; Staver et al., 2011). Tropical fires are also more abundant at higher temperatures and irradiation (Wei et al., 2020), lower soil moisture content (Chen et al., 2013), and stronger rainfall variability (Staal, van Nes, et al., 2018; Van der Werf et al., 2008). In the dry tropics, wildfires tend to be fuel limited, whereas in the wet tropics they tend to be drought limited (Pausas & Ribeiro, 2013). Possibly for the latter reason, there is a negative relation between inter‐annual rainfall variability and forest cover in the wet tropics particularly (Holmgren et al., 2013). Rainfall also increases soil moisture, which decreases fire risk in tropical forests (Chen et al., 2013). Yet, surprisingly, in seasonally flooded forests, low oxygen availability may cause fuel to accumulate in the organic soil layer, increasing forest flammability (Dos Santos et al., 2016; Flores et al., 2017). In addition to opening up canopies, forest fires also increase fuel loads by creating dead wood and partially combusted biomass, favoring subsequent fires (Cochrane et al., 1999). Forest fires can be a major source of atmospheric CO_2_, especially where forests are located on peat soils, which may turn into fuel during extreme droughts or in response to land‐use changes (Page et al., 2002; Van der Werf et al., 2010). The resulting atmospheric warming increases lightning frequency (Mariani et al., 2018), thus adding potential fire ignition sources (Ramos‐Neto & Pivello, 2000) and causing tree mortality directly (Yanoviak et al., 2020).

Feedback between forest cover and fire is relatively well established in the tropics. This double‐negative effect (thus positive feedback) may create alternative stable states between closed and open ecosystems. Although the feedback occurs at landscape scale, its consequences are observed across a range of climates and regions.

### 2.5 Interactions with soils

Soil fertility plays a fundamental role in shaping tropical forest communities (Baldeck et al., 2013) and dynamics (Quesada et al., 2012), and by increasing tree growth rates, it also affects tree cover positively (Bond, 2010; Hoffmann et al., 2012; Murphy & Bowman, 2012). In particular, total soil phosphorus (P) is known to strongly affect tree wood production rates, whereas soil exchangeable potassium seems to negatively affect wood density at the community level (Quesada et al., 2012). As a result, fertile soils are associated with fast‐growing tree species. In Africa, soil fertility may also favor growth of nutrient‐demanding native C4 grasses (Archibald & Hempson, 2016; Staver et al., 2017), but in South America, African grasses become invasive where nutrients are more abundant, whereas the native C4 grass species are often adapted to nutrient‐limited soils (Bustamante et al., 2012). Similarly, soil moisture is vital for plants, as it benefits photosynthesis, plant nutrition, and plant growth (Lambers & Oliveira, 2019; Miguez‐Macho & Fan, 2021; Sousa et al., 2020). In turn, forest trees produce litter with high nutrient content, which may improve soil nutrient availability and fertility (Chapman et al., 2006; Davidson et al., 2007; Paiva et al., 2015). Forest trees also increase soil moisture by reducing radiation and wind in the understory (Holdsworth & Uhl, 1997; Lal & Cummings, 1979), increasing infiltration (Celentano et al., 2017; Lal & Cummings, 1979), and because their roots can lift water from deep to shallow soil layers (Oliveira et al., 2005). However, as trees transpire, they reduce soil moisture (Seneviratne et al., 2010). Yet, when repeated disturbances such as shifting cultivation or wildfires keep forest cover open, they enhance topsoil erosion rates, reducing soil fertility and soil organic matter. This reduces soil infiltration capacity and soil moisture (Celentano et al., 2017; Lal & Cummings, 1979) and further slows down the tree growth that maintains forest cover (Flores et al., 2020). Soil moisture is also negatively affected in forests previously used for livestock production and logging, which cause soil compaction, reducing water infiltration, and increasing soil erosion (Batey, 2009; DeArmond et al., 2019).

Soils that are seasonally waterlogged or flooded, however, may be stressing for trees because anoxic conditions limit tree transpiration (de Parolin et al., 2004). Seasonal flooding may either increase or decrease soil fertility, depending on whether the flood waters derive from nutrient‐rich or ‐poor soils (Junk et al., 2011). Nonetheless, as shown in the Amazon, by slowing down tree growth, flooding may reduce forest recovery after disturbances, allowing the ecosystem to persist in a low tree cover state (Flores et al., 2017). Hence, if floodable areas expand with climate change, forests may be replaced by open vegetation. Furthermore, in the wet tropics, permanently inundated soils may allow the accumulation of peat (Wang et al., 2018).

On average, one‐third of all carbon stored in tropical forests is in the soil (Pan et al., 2011). Higher temperatures may accelerate soil organic matter breakdown, increasing soil respiration rates and consequently carbon emissions from soils to the atmosphere (Metcalfe et al., 2018; Nottingham et al., 2020). Soil moisture may also increase soil respiration under drier climatic conditions and decrease soil respiration when conditions are too wet (Waring & Hawkes, 2015). In the case of peatlands, which normally emit CH_4_ under permanently flooded conditions, drought intensification may reduce flooding and increase CO_2_ emissions by soil respiration (Wang et al., 2018) or fire combustion (Brando et al., 2019). In contrast, the expansion of floodable areas in tropical forests may cause mass tree mortality (Resende et al., 2020), increasing both CO_2_ and CH_4_ emissions from aerobic and anaerobic respiration, respectively (Fearnside & Pueyo, 2012; Melack et al., 2004).

Plant–soil interactions are also highly dependent on soil biota, particularly in tropical forests, where nutrients are stored in the aboveground biomass (Wardle et al., 2004) and in the form of topsoil detritus. This implies that microbial processes are fundamental to maintain nutrient cycling (Camenzind et al., 2018; Lambers & Oliveira, 2019; Moore et al., 2004). Symbiosis between trees and symbiotic fungi enhances nutrient uptake and defense against pathogens, giving a competitive advantage to host trees, particularly on nutrient‐poor soils (Corrales et al., 2018; Peh et al., 2011). Although ectomycorrhizal fungi seem to be negatively affected by soil fertility, as they are often more abundant and diverse in nutrient‐poor soils (Corrales et al., 2018), most mycorrhizal fungi are benefited by P addition (Camenzind et al., 2018). Hence, soil fertility effects on symbiotic fungi can vary, depending also on the composition of host trees. Moreover, ectomycorrhizal fungi are known to slow down tree litter decomposition, affecting nutrient availability for other microorganisms and plants (McGuire et al., 2010). Therefore, in hyperdominated tree communities, which are common across tropical forests (Ter Steege et al., 2013), forest cover stability may largely depend on a few host tree species and their interactions with symbiotic fungi.

Nitrogen enters the tropical forest mainly through atmospheric dinitrogen (N_2_) fixation by symbiotic and free‐living microorganisms (Hedin et al., 2009). Usually, in undisturbed forests, nitrogen is abundant in the organic topsoil, but after disturbances that remove or burn topsoils, nitrogen can become limiting, until its concentration recovers along with secondary succession (Davidson et al., 2007; Pellegrini et al., 2018). Although symbiotic N_2_ fixation may contribute to increase carbon accumulation in secondary forests (Levy‐Varon et al., 2019), after fire, N_2_ fixation can be reduced (Bomfim et al., 2020). Soil phosphorus and soil moisture can be strong limiting factors of N_2_ fixation rates in tropical forests (Van Langenhove et al., 2020), implying that if extreme drought events and disturbances by fire and deforestation happen more often, intensifying soil erosion (Borrelli et al., 2017; Flores et al., 2020), N availability may severely decrease. This process may also reduce atmospheric CO_2_ fertilization of tropical forests, which is limited by N and P (Terrer et al., 2019). Nonetheless, recent evidence suggests that atmospheric N and P fertilization may occur due to biomass burning of tropical ecosystems, which could potentially compensate for N and P losses in disturbed forests (Bauters et al., 2018, 2021).

Overall, interactions involving tropical forest soils occur at the local and landscape scales. Feedbacks are relatively known in undisturbed forests, yet our understanding of the impacts of global changes, such as extreme weather events and fires, on these processes is constantly being advanced by recent studies, revealing potentially novel connections.

### 2.6 Interactions with fauna

Animals can be highly dependent on forest cover, with many species being sensitive to open habitats (Banks‐Leite et al., 2014; Barlow et al., 2016; Laurance et al., 2004; Pfeifer et al., 2017). In turn, animals affect tropical forest cover via trophic and mutualistic networks of interactions.

Animals influence trophic networks through top‐down processes. Carnivores control populations of other animals, including herbivores, frugivores, and pollinators (Ripple et al., 2014) and their loss may have large indirect effects on ecosystem processes, such as primary productivity and erosion control (Estes et al., 2011). In the forests of Venezuela, the loss of carnivores allowed populations of monkeys and leaf‐cutter ants to grow and overbrowse tree saplings, reducing tree recruitment rates, thus risking loss of forest persistence (Terborgh et al., 2001). In the African Serengeti, browsers, such as elephants, feed from woody plants and may negatively affect tree cover by reducing tree recruitment (Sinclair et al., 2007). Although elephants reduce tree density in African tropical forests, they may also select for large dense‐wood trees, potentially increasing carbon storage capacity in those ecosystems (Berzaghi et al., 2019). In contrast to browsers, wildebeest grazers reduce grass biomass and hence landscape flammability, indirectly facilitating the expansion of forest cover (Sinclair et al., 2007; Staver et al., 2021). Thus, herbivores may affect forest cover in contrasting manners (Archibald & Hempson, 2016; Hempson et al., 2015; Van Langevelde et al., 2003); in general, browsers negatively affect forest cover, whereas grazers have similar effects on grasses, reducing tree–grass competition and wildfires, indirectly promoting forest cover (Staver et al., 2021).

Tropical forest animals also influence trophic networks through bottom‐up processes. By moving across the landscape, large‐ and medium‐sized vertebrates (mainly carnivores, herbivores, and frugivores) transport considerable amounts of nutrients from nutrient‐rich areas, such as floodplains, into nutrient‐poor forests, mainly through their dung and bodies (Doughty et al., 2013; Metcalfe et al., 2014). Plant consumption by browsers may reduce nutrient fluxes from trees to the soil at the local scale, yet these mobile animals often contribute to deposit organic matter and accelerate carbon and nutrient cycling across broader areas (Metcalfe et al., 2014). This process of landscape nutrient enrichment by mobile animals is thought to have influenced forest dynamics and tree species distributions over millennia. Yet, with the loss of mega‐fauna species, this process has been fading, potentially altering tropical forest cover at continental scales (Gardner et al., 2019).

Mutualistic networks of interactions between plants and animals, in particular seed dispersal and pollination, are also critical for tropical forests. By dispersing tree seeds, mobile animals accelerate the recovery of disturbed patches (Lundberg & Moberg, 2003; McConkey et al., 2012). In wet tropical forests, 82% of all tree species depend on seed dispersal by frugivores (Howe & Smallwood, 1982). Frugivores may also facilitate forest cover expansion over savannas, as shown in the northern edge of the Amazon where large tapirs travel long distances and disperse tree seeds within forest fragments (Fragoso et al., 2003). When frugivores disappear, dispersal limitations may cause widespread collapse of tree populations (Gardner et al., 2019; Peres et al., 2016). Pollination is another critical mechanism for tree sexual reproduction and recruitment. It is estimated that 94% of all tropical plants depend on animal pollination to reproduce (Ollerton et al., 2011), implying that pollinators positively affect resource availability for frugivores. Consequently, the loss of pollinators due to deforestation and habitat fragmentation, for instance, may arrest forest recovery and potentially reduce forest cover (Neuschulz et al., 2016).

Animals may also accelerate the spread of disturbances, such as diseases, fire, and alien plant invasion, reducing the resilience of forest landscapes to systemic collapse (Lundberg & Moberg, 2003). For instance, in tropical forests of northern Australia, hawks intentionally spread fires to flush out their prey (Bonta et al., 2017). Large wildfires, in turn, may harm animals and cause widespread mortality (Barlow et al., 2016). The spread of alien invasive plants by birds and mammals into forested areas is another example, such as in rainforests of Madagascar where non‐native guava trees are invasive, partly due to lemur dispersal (DeSisto et al., 2020). Even carnivores can spread invasive plants by moving across disturbed landscapes (Hämäläinen et al., 2017).

In summary, interactions involving animals in tropical forests involve multiple feedback loops, mostly at the local and landscape scales. Outcomes of these interactions can promote forest cover when they facilitate tree establishment after disturbances, or downgrade forest cover when they facilitate the spread of disturbances.

### 2.7 Interactions with humans

Modern humans have interacted with tropical forests for at least 13 kya in South America, 45 kya in Southeast Asia, and at least 200 kya in Africa, and as a result, our influences can be found in all landscapes at varying degrees, even in the remotest regions (Levis et al., 2017; Roberts et al., 2017). Tropical forest societies accumulated a profound knowledge of ecological processes and functions over generations (Berkes et al., 2000), which, in turn, made them highly dependent on the forest (Cámara‐Leret et al., 2019). More recently, since the Industrial Revolution (1800s) and particularly after the Great Acceleration (1950s) (Steffen et al., 2015), humans started to transform tropical forest ecosystems more intensely and faster than in the previous millennia, introducing new forms of management and land uses, and persistently altering key ecological interactions (Malhi et al., 2014). Here we divide these human–forest interactions into two groups: one related to the ancient interactions with indigenous peoples and local communities (IPLCs), and another related to the recent transformations by globalized societies.

For a long time, ecologists have understood tropical forests as natural, pristine ecosystems, where human intervention had been minimal (Willis et al., 2004). Yet, in recent decades, studies have revealed extensive signs of ancient human use and management of tropical forest landscapes (Levis et al., 2017; Roberts et al., 2017). This long‐term human agency has re‐shaped plant–soil interactions, for instance by increasing soil fertility and forest productivity (Levis, Peña‐Claros, et al., 2020), or by decreasing soil fertility due to erosion (Flores et al., 2020). It has re‐shaped plant–animal interactions through overhunting that reduced seed dispersal and nutrient spread (Doughty et al., 2013). However, traditional landscape management may also increase the availability of fruit trees and, therefore, resources that increase frugivore density (Levis et al., 2018). IPLC's forest management may decrease the density of tree species useful for construction, but often they increase the density of edible arboreal species (Levis, Peña‐Claros, et al., 2020). IPLCs may also reduce forest cover locally through small‐scale deforestation (Jakovac et al., 2016). Nonetheless, these populations often contribute to reduce deforestation, logging and fires at regional scales, contributing to maintain forest cover (Asner et al., 2005; Nepstad et al., 2006). Even practices that often reduce forest cover, such as traditional fire use, have been applied in ways that did not cause major forest collapse (Willis et al., 2004). Not surprisingly, forests that are still being used and managed by IPLCs are often better protected worldwide (Garnett et al., 2018). In the Amazon, forest cover is clearly better protected inside indigenous territories and protected areas when compared to surrounding areas (Nepstad et al., 2006), causing a ninefold decrease in carbon emissions (Walker et al., 2020).

Today, globalized societies use and manage tropical forests in an entirely new fashion (Barlow et al., 2018; Malhi et al., 2014). Industrial activities have transformed these ecosystems into monoculture plantations and other forms of land use, disrupting ancient ways of living, of ecological processes, and of ecosystem functions, and increasing greenhouse gas emissions. The main drivers of tropical forest loss are livestock production in South America, shifting agriculture in Africa, and palm‐oil plantations in Asia (Barlow et al., 2018; Curtis et al., 2018; Malhi et al., 2014; Qin et al., 2019). In the case of livestock, this activity contributes to methane emissions (Saunois et al., 2020), and also to introducing alien invasive C4 grasses in the system. Invasive grasses can spread into disturbed forests (Barlow et al., 2018; D'Antonio & Vitousek, 1992; Malhi et al., 2014), where they compete with recruiting trees, reducing forest cover (Hoffmann et al., 2004) particularly when the soil is more fertile (Bustamante et al., 2012; Penuelas et al., 2010). Livestock browsing, trampling, and soil compaction further contribute to reducing forest cover. Yet, because livestock reduces C4 grass biomass, it may indirectly benefit forest cover by reducing fire risk (Bernardi et al., 2019). In contrast, invasive trees may not necessarily change forest cover, but by excluding native tree species, they alter forest structure and composition (Asner et al., 2008). Remaining forests are increasingly degraded by human activities, such as logging, mining, and road construction, which open forest cover for C4 grasses and alien invasive plants that promote wildfires and soil erosion; all of which further reduce forest cover (Alamgir et al., 2019; Alencar et al., 2004; Barlow et al., 2018; Fine, 2002; Holdsworth & Uhl, 1997; Malhi et al., 2014; Siegert et al., 2001; Silvério et al., 2013; Veldman & Putz, 2011). Logging roads also expand human access to standing forest, increasing deforestation rates (Kleinschroth et al., 2019) and the dispersal of alien invasive grasses (Veldman & Putz, 2010). Because timber volume hardly recovers in time for continuous harvest cycles, industrial logging in tropical forests probably has a negative effect on itself, as well as a positive effect on greenhouse gas emissions (Zimmerman & Kormos, 2012). Increasing wildfire regimes not only threaten forest cover and biodiversity (Brando et al., 2019; Kelly et al., 2020), but also contribute to perpetuate poverty and dependence on non‐traditional fire use (Cammelli et al., 2020). This negative effect of fires on tropical forests is further accentuated by rainfall variability, with droughts facilitating the use of fire for clearing dead biomass, thus increasing deforestation rates in synergy with direct human effects (Staal, Flores, et al., 2020). Previously burnt forests are not only more likely to burn again (Alencar et al., 2004; Cochrane et al., 1999), but also to be deforested (Qin et al., 2019). Similarly, abandoned secondary forests are more likely to be cleared than adjacent primary forests, implying that deforestation may have a positive effect on itself (Wang et al., 2020).

In sum, although indigenous peoples and local communities may slightly reduce forest cover near their homes, compared to globalized societies, they have a net positive effect on forest cover at much broader scales, as revealed by field and satellite observations (Garnett et al., 2018; Nepstad et al., 2006).

## 3 EMERGING FEEDBACKS

### 3.1 A network of interactions

**FIGURE 2:**
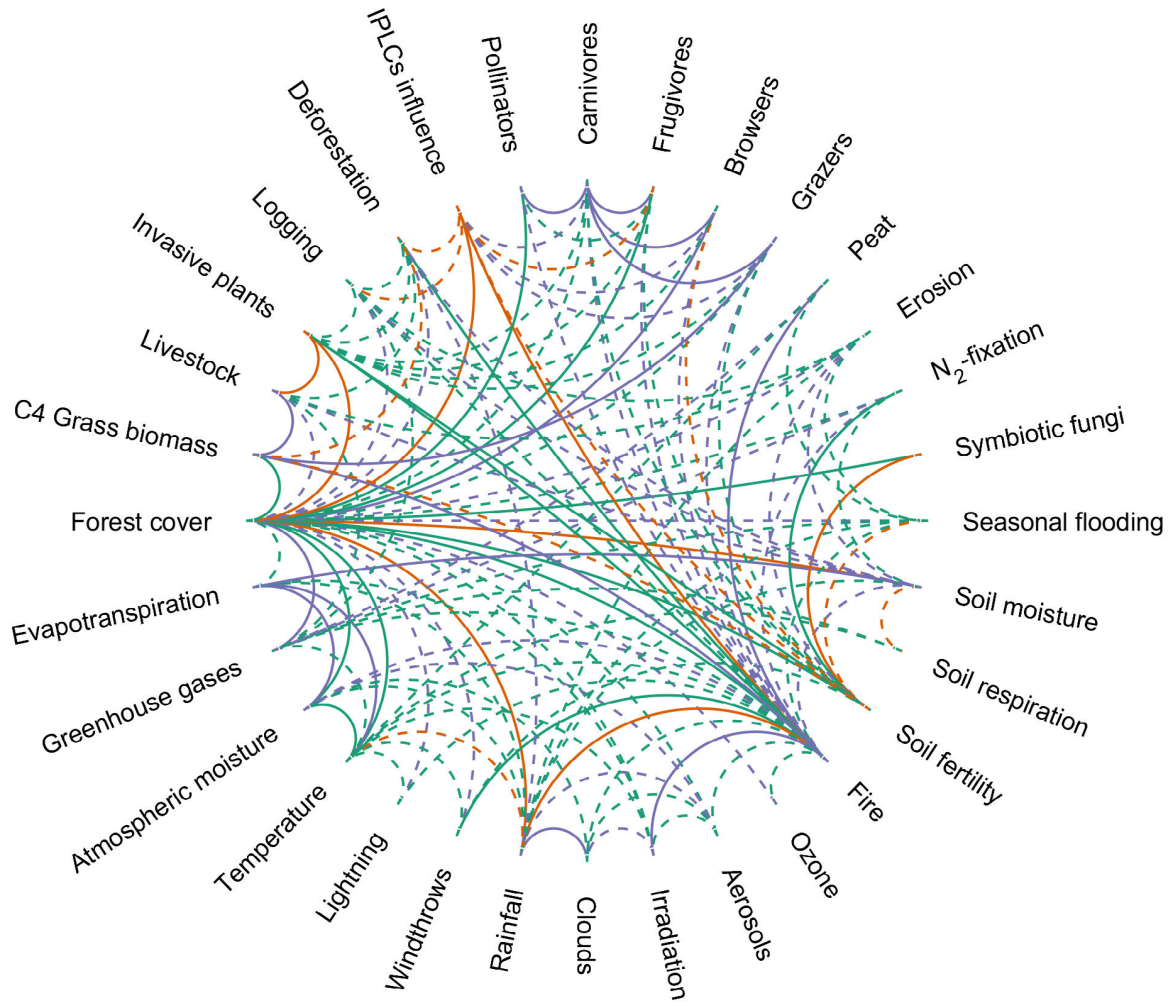
A tropical forest network of interactions and feedbacks. Solid lines indicate feedbacks between the two connected variables and dashed lines indicate one‐way interactions between them. Green indicates a positive feedback or interaction, purple a negative feedback or interaction, and brown a positive and/or negative feedback or interaction (e.g., depending on the spatial scale of analysis). Evidence for some interactions and feedbacks may come from particular spatial and temporal contexts (e.g., a specific continent; see Table 2), but because of their assumed generality, we show them as a single tropical forest network. All lines correspond to processes discussed in the main text (Section 2), and shown in Table 1. For the direction of each interaction, see Table 1. IPLCs influence refers to the relative influence of indigenous peoples and local communities compared to that of globalized societies. For details on the effects, scales and references of one‐way interactions (dashed lines), see Table S1. IPLC, indigenous peoples and local communities.

In our literature review, we identified interactions among 32 components of tropical forests, including social, biological, physical, and biogeochemical aspects of the system (Table 1). These components can be thought of, and visualized as, elements in a tropical forest network (Figure 2). Some elements of this network represent stocks and can be understood as state variables, for example soil moisture content. However, other elements are processes, for example deforestation. Despite this loose definition of eligible network elements, all of them were chosen to be in principle quantifiable, and defined such that they interact with more than one other element. Although we recognize the diversity in functioning and effects of different species and processes on tropical forests, where possible, we simplified them to facilitate this broad overview of interactions. In total, we identified 170 individual interactions from the scientific literature. Even though we realize that this set of interactions will be incomplete, it nonetheless unveils myriad previously unidentified causal pathways in tropical forests.

**TABLE 1:** Interactions in tropical forests, including the social, biological, physical, and chemical dimensions of the system. We identified 170 individual interactions among 32 components, some of which form direct feedbacks, whereas other ones may form feedback loops involving more than two components. Causes are shown in rows and effects in columns, with green for positive effects, purple for negative effects, and brown when both positive and negative effects are possible, depending on the case (e.g., the spatial scale or region). For more details on the effects, scales and references for each interaction (see Table S1)

	Forest cover	Evapotranspiration	Greenhouse gases	Atm. moisture	Temperature	Lightning	Windthrows	Rainfall	Clouds	Irradiation	Aerosols	Ozone	Fire	Soil fertility	Soil respiration	Soil moisture	Seasonal flooding	Symbiotic fungi	N_2_‐fixation	Erosion	Peat	Grazers	Browsers	Frugivores	Carnivores	Pollinators	IPLCs influence	Deforestation	Logging	Invasive plants	Livestock	C4 Grass biomass
Forest cover																																
Evapotranspiration																																
Greenhouse gases																																
Atm. moisture																																
Temperature																																
Lightning																																
Windthrows																																
Rainfall																																
Clouds																																
Irradiation																																
Aerosols																																
Ozone																																
Fire																																
Soil fertility																																
Soil respiration																																
Soil moisture																																
Seasonal flooding																																
Symbiotic fungi																																
N_2_‐fixation																																
Erosion																																
Peat																																
Grazers																																
Browsers																																
Frugivores																																
Carnivores																																
Pollinators																																
IPLCs influence																																
Deforestation																																
Logging																																
Invasive plants																																
Livestock																																
C4 Grass biomass																																

Abbreviation: IPLC, indigenous peoples and local communities.

When these interactions form a feedback or a feedback loop, they can change the forest in unexpected and abrupt ways. Negative feedbacks are stabilizing as they dampen change, whereas positive feedbacks are self‐reinforcing and sometimes propel the ecosystem to a contrasting state (DeAngelis et al., 1986; Van Nes et al., 2016). Our findings reveal an unexplored world of feedbacks that influence the dynamics of tropical forests. They indicate how the distribution of forests and species along environmental gradients is not simply a result of deterministic effects (Whittaker, 1967), because feedbacks transform both the ecosystem and its environment over long time scales. Feedbacks have influenced the evolution of tropical forest biodiversity, as species transform their habitat and re‐construct their niche (Laland et al., 2000). Hence, to understand how tropical forests may respond to today's rapidly changing environment, it is necessary to identify potential positive and negative feedback loops in the system.

In Table 2, we highlight some examples of feedbacks and feedback loops identified in our review. For instance, a classical example involves mutual negative interactions between forest cover and fire, in which one suppresses the other, forming a positive feedback that contributes to stabilize high and low tree cover as alternative stable states across the tropics (Staver et al., 2011; Van Nes et al., 2018). The positive feedback between IPLCs and forest cover is another example, in which their mutual positive interactions contribute to keep tropical forests resilient (Garnett et al., 2018; Nepstad et al., 2006). We also identified novel feedback loops that might influence tropical forest functioning, yet their resulting effects remain unknown (Table 2). For example, fires emit greenhouse gases, increasing atmospheric temperature, which increases lightning and fire risk. In theory, this positive feedback loop could intensify fire regimes in remote regions where lightning is the main ignition for wildfires (Kelley et al., 2019; Ramos‐Neto & Pivello, 2000).

**TABLE 2:** Examples of feedbacks and feedback loops reported in the literature and in this review

Feedback components	Type	Description	Spatial scale	Evidence	Status
Forest–carbon–global climate	Positive loop	Forests store carbon, reducing global warming, climatic variability and forests more resilient	Global	Theoretical, experimental, observational	Literature
Forest–vapor–rainfall	Positive loop	Forest cover increases atmospheric vapor and thus rainfall, increasing forest cover	Regional	Theoretical, observational	Literature
Forest—fire	Positive	Forest cover decreases fire risk. Fire decreases forest cover	Local	Theoretical, experimental, observational	Literature
Forest–soil erosion–soil fertility	Positive loop	Forest cover decreases soil erosion. Erosion decreases soil fertility, decreasing forest growth	Local	Theoretical, experimental, observational	Literature
Forest–soil biota–soil fertility	Positive loop	Forest litter increases soil biota, nutrient cycling, soil fertility and hence forest growth	Local	Theoretical, experimental, observational	Literature
Drought–fires–peat–global warming	Positive loop	Droughts increase fire that destroys peat forest, emitting carbon that increases global warming and droughts	Local to global	Theoretical, observational	Literature
Grazers–fires–forest	Negative loop	Animal grazers decrease grass biomass, decreasing fires, which allows forest growth, decreasing grasses and consequently grazers	Local	Theoretical, experimental, observational	Literature
Mobile animals–nutrients–forest	Positive loop	Mobile animals move across landscapes, transporting nutrients through their dung and bodies, increasing soil fertility and forest growth	Local to regional	Theoretical, observational	Literature
IPLCs–forest	Positive	Indigenous peoples and local communities protect the forest, increasing forest cover. Forest cover increases resources for IPLCs	Regional	Theoretical, observational	Literature
Drought–deforestation	Positive	Droughts increase deforestation by facilitating fire use. Deforestation decreases forest cover and rainfall recycling, increasing drought	Regional	Theoretical, observational	Literature
Windthrows–fires–forest	Positive loop	Forest cover loss by windthrows increase fire risk, keeping the forest vulnerable to fires and winds	Local	Theoretical	This review
Deforestation–pollinators–frugivores–forest	Positive loop	Deforestation leads to fragmentation, decreasing pollinators, resulting in fewer fruits, decreasing seed dispersal by frugivores and forest cover	Local to regional	Theoretical	This review
Fire–greenhouse gases–temperature–lightning	Positive loop	Fires emit greenhouse gases, increasing atmospheric temperature, which increases lightnings and fire risk	Regional	Theoretical	This review
Drought–fire–N_2_‐fixing–soil fertility–forest	Positive loop	Drought and fire decrease N_2_ fixation, decreasing soil fertility and forest growth. Less forest cover increases drought and fire	Local	Theoretical	This review
Animals–invasive grasses–disturbances–forest	Negative loop	Mobile animals disperse invasive grasses, decreasing native plants and increasing fires, thus decreasing forest cover and habitat for animals	Local to regional	Theoretical	This review

Abbreviation: IPLC, indigenous peoples and local communities.

### 3.2 Case studies

To illustrate how feedbacks emerge from our network, we highlight three selections out of this web of interactions in Figure 2, which function as arbitrarily chosen, but illustrative, case studies. We consider one example from each tropical continent: South America, Africa, and Asia.

#### 3.2.1 South America: Wildfires in the Pantanal wetlands

**FIGURE 3:**
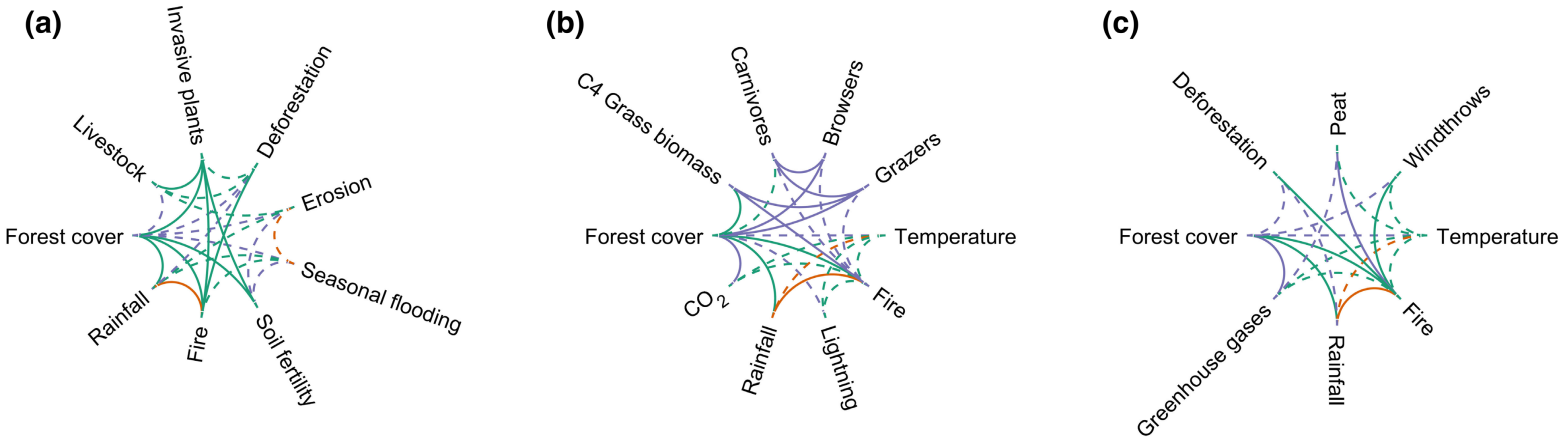
Interactions and feedbacks in tropical forests of three continents (a) South America, (b) Africa, and (c) Asia. Each study case is a selection of the network of interactions shown in Figure 2, adjusted for the specific contexts discussed, to illustrate how feedbacks can emerge from our network. We consider one example from each tropical continent to address a timely and relevant research topic.

This network example (Figure 3a) comprises nine interacting elements at multiple scales, connecting the Amazon and Pantanal regions of South America, the world's largest tropical forest and wetland savanna biomes, respectively. In the Amazon, rainfall is recycled by the forest's evapotranspiration, a process that contributes to rainfall in the Pantanal region (Staal, Tuinenburg, et al., 2018). Deforestation of the Amazon forest, however, is disrupting this continental scale interaction, reducing rainfall particularly during the dry season (Staal, Tuinenburg, et al., 2018). In the floodplain savannas of the Pantanal, less rainfall implies disturbance to the seasonal flooding regime, exposing landscapes to the risk of large wildfires (Mega, 2020). Moreover, locally, the Pantanal region is increasingly under pressure from livestock production, which introduces invasive non‐native grasses that further increase landscape flammability (D'Antonio & Vitousek, 1992). Large wildfires reduce forest cover within the savanna landscape (Flores et al., 2021) and increase landscape vulnerability to flood erosion, reducing soil fertility and further contributing to loss of forest cover (Flores et al., 2020). Local clearing of the native Pantanal vegetation for livestock production also contributes to accelerate soil erosion and the risk of wildfires (de Oliveira et al., 2014; Guerra et al., 2020). Although invasive grasses may have a positive effect on topsoil fertility where soils are nutrient limited, this mechanism usually favors the persistence of invasive grasses (D'Antonio & Vitousek, 1992). Therefore, continued deforestation in the Amazon could expose the Pantanal landscapes to unprecedented wildfire regimes, threatening the great biodiversity of this iconic region, as observed in the 2020 fire season (Mega, 2020).

#### 3.2.2 Africa: Forest encroachment in savanna landscapes

This network example (Figure 3b) comprises 10 interacting elements, which operate at local to regional scales and are particularly relevant to African forest–savanna transition zones. Ongoing global emissions of CO_2_ are predicted to cause forest expansions in African savannas. This can be due to both a direct effect through CO_2_ enrichment and indirectly via the global climate. Climate‐change scenarios indicate that rainfall increases may cause an expansion of the area suitable for forests (Staal, Fetzer, et al., 2020). Indeed, encroachment of forests into savannas is ongoing (Venter et al., 2018). From our identified interactions emerges an interacting set of feedbacks that may affect this process of forest encroachment, now and in the future. From Figure 3b emerges an interplay between the global climate, and animal and fire ecology, set in motion by anthropogenic emissions of greenhouse gases. With rising atmospheric CO_2_ concentrations, forest cover increases directly, but also indirect influences can be identified: with increasing temperatures, the frequency of lightning strikes and consequently that of fires is increased. These fires predominantly occur in the savannas, where many large grazers are still present. Grazers and fire both “compete” for grasses (Bond & Keeley, 2005). As a result, despite its negative effect on forest cover, an increase in fires can in theory strongly suppress grazer abundance. This would reduce food availability for carnivores, which can be predicted to shift their diet toward browsers. The resulting browser suppression could expand forest cover even further, given that these animals eat juvenile trees.

#### 3.2.3 Asia: Synergistic threats to the peatland forests of Borneo

This network example (Figure 3c) comprises eight interacting elements, at various spatial scales, with a potentially important positive feedback loop to the Earth's climatic system. With increasing concentration of greenhouse gases in the atmosphere (CO_2_ and CH_4_), global temperature is rising, causing El Niño‐related droughts in Southeast Asia (Page et al., 2002; Wang et al., 2019). These droughts increase the risk of wildfires that penetrate peatland forests (Page et al., 2002; Turetsky et al., 2015). Deforestation in the region also contributes to desiccating peat soils, increasing their flammability. In addition, higher atmospheric temperatures due to global warming could increase the frequency and severity of wet extremes and thereby the intensity of windthrows (as observed in the Amazon; Aleixo et al., 2019), further reducing forest cover and increasing landscape flammability. Experimental evidence (also from the Amazon forest) shows that in burnt forests with open structure, tree mortality from windthrows is higher than in unburnt mature forests (Silvério et al., 2019). In turn, windthrows reduce forest cover and increase fuel loads in the forest (Dos Santos et al., 2016; Negrón‐Juárez et al., 2010), increasing flammability and wildfire risk. It is possible that also in Borneo these combined effects may accelerate forest degradation. With more wildfires consuming peat and forest biomass, coupled with more tree mortality due to windthrows, emitting huge amounts of carbon to the atmosphere (Page et al., 2002; Turetsky et al., 2015), this example illustrates how peatlands of Southeast Asia could self‐reinforce global warming.

### 3.3 Prospects for future research

Our review is a small step in the grand challenge of understanding tropical forest functioning in the Anthropocene. The network in Figure 2, therefore, is an incomplete image, revealing numerous opportunities for future research. Below, we propose a list of open research questions that may help filling these gaps:

- What is the strength of each feedback? Quantifying the strength of feedbacks under various contexts and across scales will reveal which ones may become more important in the Anthropocene, allowing societies to manage those feedbacks and avoid abrupt transitions (Folke et al., 2010).
- Which feedbacks are more manageable to enhance tropical forest resilience? Identifying which critical feedbacks are more easily manageable can be extremely useful for practitioners and decision‐makers to increase tropical forest resilience to global changes (Biggs et al., 2012).
- How can we manage feedbacks to boost tropical forest restoration? Applying our understanding of feedbacks can inform efforts on tropical forest restoration, since many cases require intervention to break degrading feedbacks and restore the original ones.
- How can we manage feedbacks to increase tropical forest productivity for local people? Investigating ancient and novel feedbacks that increase tropical forest productivity can help enhance the availability of vital resources for local human societies (e.g., Flores & Levis, 2021).
- What do networks of other ecosystems look like, compared to those of tropical forests? Which feedbacks are present and which are absent?

## 4 CONCLUSIONS

The case studies examples in Section 3.2 are snippets of the wealth of possible interactions and feedbacks that our review (Figure 2) indicates. They serve to highlight how our approach can aid in making sense of the complexity of tropical forests to guide future research and management. Our review focused on the qualitative nature of the interactions and feedbacks in tropical forests and ignored their relative strengths, even though the quantitative balance between strong and weak positive and negative feedbacks will determine how global changes will affect tropical forests in the future. Disentangling that balance will involve developing both simple and complex mathematical models, as well as much empirical research on the individual strengths of interactions, and exploring statistical methods to study causal pathways in complex systems (Rocha et al., 2018; Sugihara et al., 2012). Although we identified a large number of interactions, a look at Table 1 also makes clear that many interactions may *not* be identified (blank fields). In some cases, these will simply not exist. In others, we may have missed them in our review, or they may not *yet* be known; hence, we welcome experts in the various particular fields to contribute to the body of knowledge that our network in Figure 2 illustrates. This may guide future research on the fascinatingly complex interplay between tropical forest structure and species, and their biophysical and socio‐ecological environment, in a changing world.

## AUTHOR CONTRIBUTIONS

Bernardo M. Flores and Arie Staal equally designed the study, reviewed the literature, and wrote the manuscript.

## CONFLICT OF INTEREST

The authors declare no conflict of interest.

## FUNDING INFORMATION

BMF is supported by Instituto Serrapilheira (Serra‐1709‐18983). AS is supported by the Talent Program grant VI.Veni.202.170 by the Dutch Research Council (NWO).

## Supporting information


Table S1.

## Data Availability

Data sharing is not applicable to this article as no new data were created or analyzed in this study.
